# Supplementary figures and images for: Partial inhibition of class III PI3K VPS-34 ameliorates motor aging and prolongs health span
Source: PLoS Biol. 2023 Jul 11;21(7):e3002165. doi: 10.1371/journal.pbio.3002165 (PMC10335676; doi:10.1371/journal.pbio.3002165)

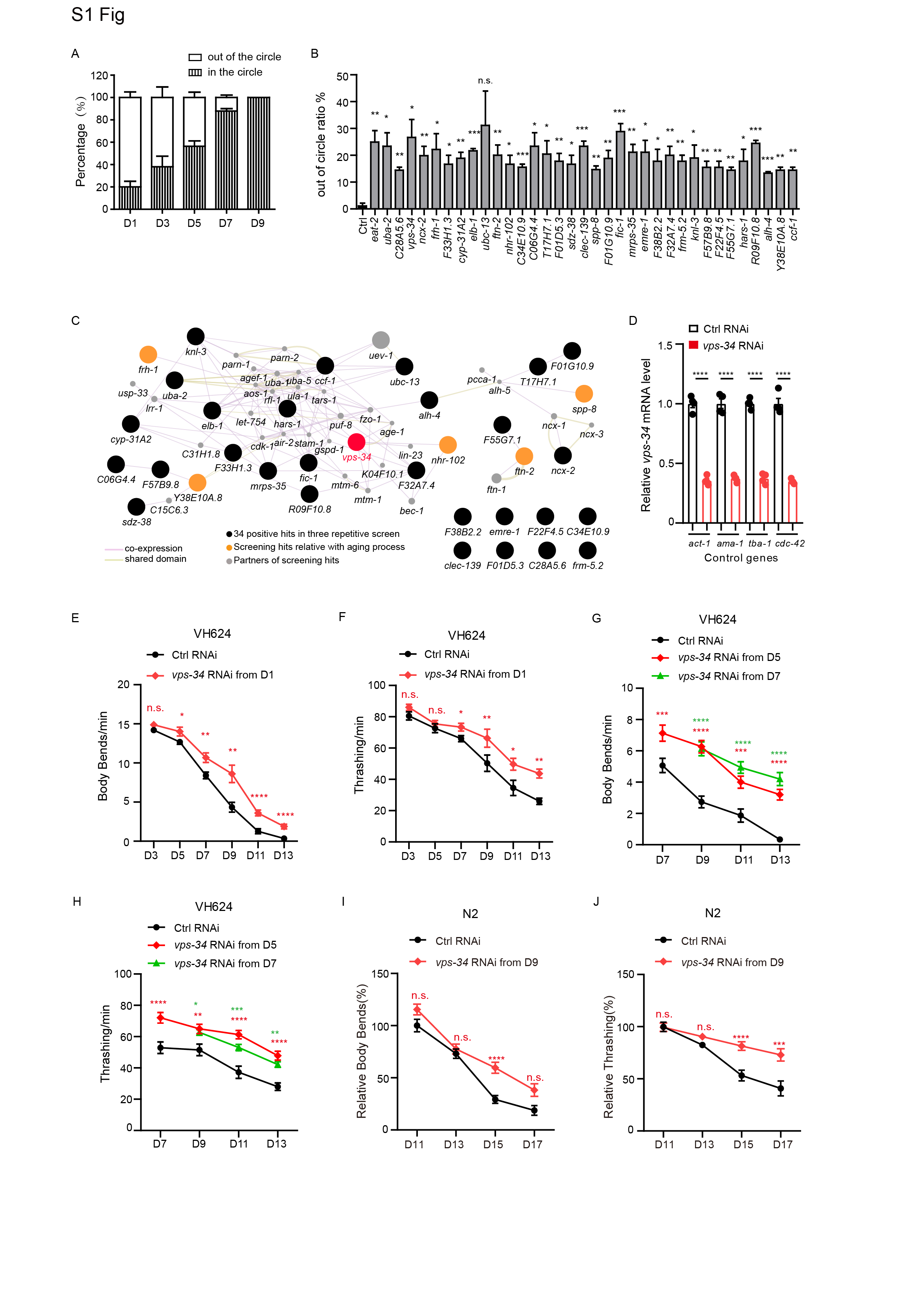

Supplement: S1 Fig — (A) The percentage of VH624 worms that stayed inside or moved outside the circle at multiple time points during aging; 30–40 worms per group. n = 3 independent experiments. (B) Summarized D9 out-of-circle ratios for ctrl and 34 positive hits. eat-2 RNAi was used as the positive control. (C) Co-expression network of identified positive genes and their partners. The network was constructed by GeneMANIA. Black and gray dots show the positive hits and their partners. Orange dots show the positive hits with related function in aging study. (D) qPCR comparing the transcription of vps-34 in vps-34 and control RNAi worms. The normalization control genes were used as act-1, tba-1, ama-1, cdc-42. (E–H) Motor behavior assays (Body bends, E and G; Thrashing, F and H) comparing ctrl and vps-34 RNAi using VH624 worms at multiple time points during aging. RNAi started from D1 (E and F) or D5 and D7 (G and H). Fifteen worms per group. n = 3 independent experiments. (I and J) Relative motor functions (Body bends, I; Thrashing, J) comparing ctrl and vps-34 RNAi using N2 worms at multiple time points during aging. Control RNAi D11 was set as 100%. RNAi started from D9. Fifty worms per group from D11 to D15, while the group of D17 scored over 20 animals. Error bars, SEM. *P < 0.05, **P < 0.01, ***P < 0.001, ****P < 0.0001. n.s., not significant. B, D, Unpaired two-tailed Student’s t test. E, F, I, J, Two-way ANOVA with Sidak’s multiple comparisons test. G, H, Two-way ANOVA with Dunnett’s multiple comparisons test. Raw data can be found in the Supporting information (S1 Data). (TIF) [file pbio.3002165.s001.tif]

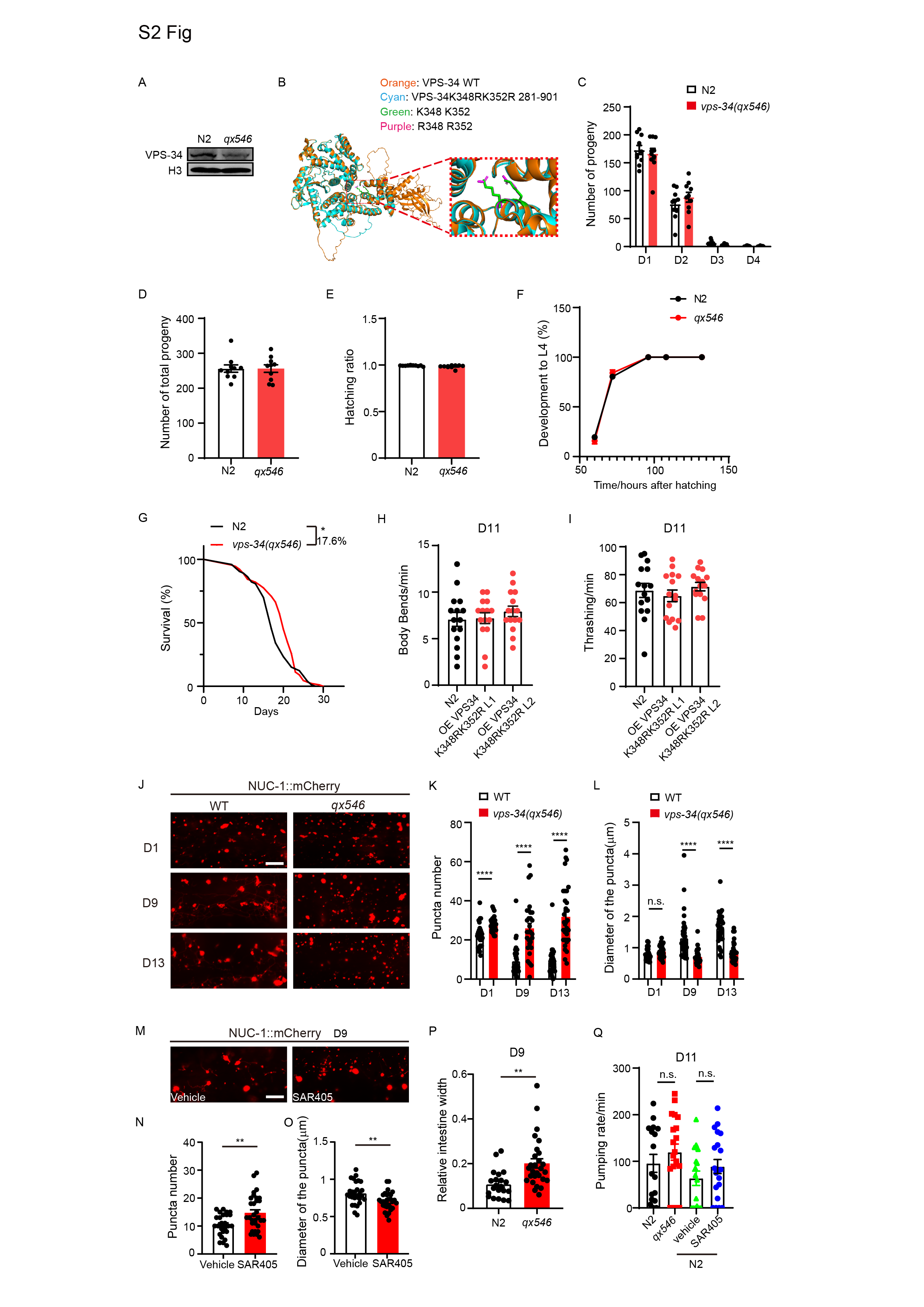

Supplement: S2 Fig — (A) Western blot shows the protein levels of VPS-34 in wild-type (N2) and qx546 worms. (B) Protein structures of wild-type (WT) and mutant (qx546, K348RK352R) predicted by AlphaFold2 using root-mean-square-deviation (RMSD) of 0.412 Å between 519 pairs of atoms. Orange: full-length WT VPS-34; Cyan: 281–901 of VPS-34 K348RK352R mutant, which overlaps well with wild-type; Green: WT K348 and K352 sites; Purple: Mutant R348 R352 sites. (C) Quantification of progeny numbers at D1, D2, D3 and D4 of adulthood in N2 and qx546 worms. n = 10 worms per genotype. (D) Total brood size in N2 and qx546. n = 10 worms per genotype. (E) The hatching ratio of eggs in wild-type (N2) and vps-34(qx546) worms. Progeny from 10 worms were calculated per genotype. (F) The proportion of N2 and qx546 worms that developed to L4 stage at different time points, starting from 60 h after hatching. Progeny from 10 worms were calculated per genotype. (C–F) n = 2 independent experiments. (G) Survival analysis of N2 and vps-34(qx546). n = 3 independent experiments. (H and I) Motor behavior assays (Body bends, H; Thrashing, I) comparing ctrl and VPS-34 K348RK352R OE in N2 worms at D11. Two transgenic lines (L1, L2) were tested. Fifteen worms per group. n = 2 independent experiments. (J) Representative images showing the lysosomal reporter NUC-1::mCherry in the epidermis of WT and vps-34(qx546) worms at D1, D9, and D13. Scale bar, 10 μm. (K) Quantification of NUC-1::mCherry puncta numbers in the epidermis of WT and vps-34(qx546) at D1, D9, and D13. Over 25 worms per group. (L) Quantification of NUC-1::mCherry puncta diameter in the epidermis of WT and vps-34(qx546) at D1, D9, and D13. Over 25 worms per group. (M) Representative images showing the lysosomal reporter NUC-1::mCherry in the epidermis of vehicle or SAR405-treated worms at D9. Scale bar, 10 μm. (N and O) Quantification of NUC-1::mCherry puncta numbers (N) and diameter (O) in the epidermis of vehicle or SAR405-treated worms at D9. Over 25 worms [file pbio.3002165.s002.tif]

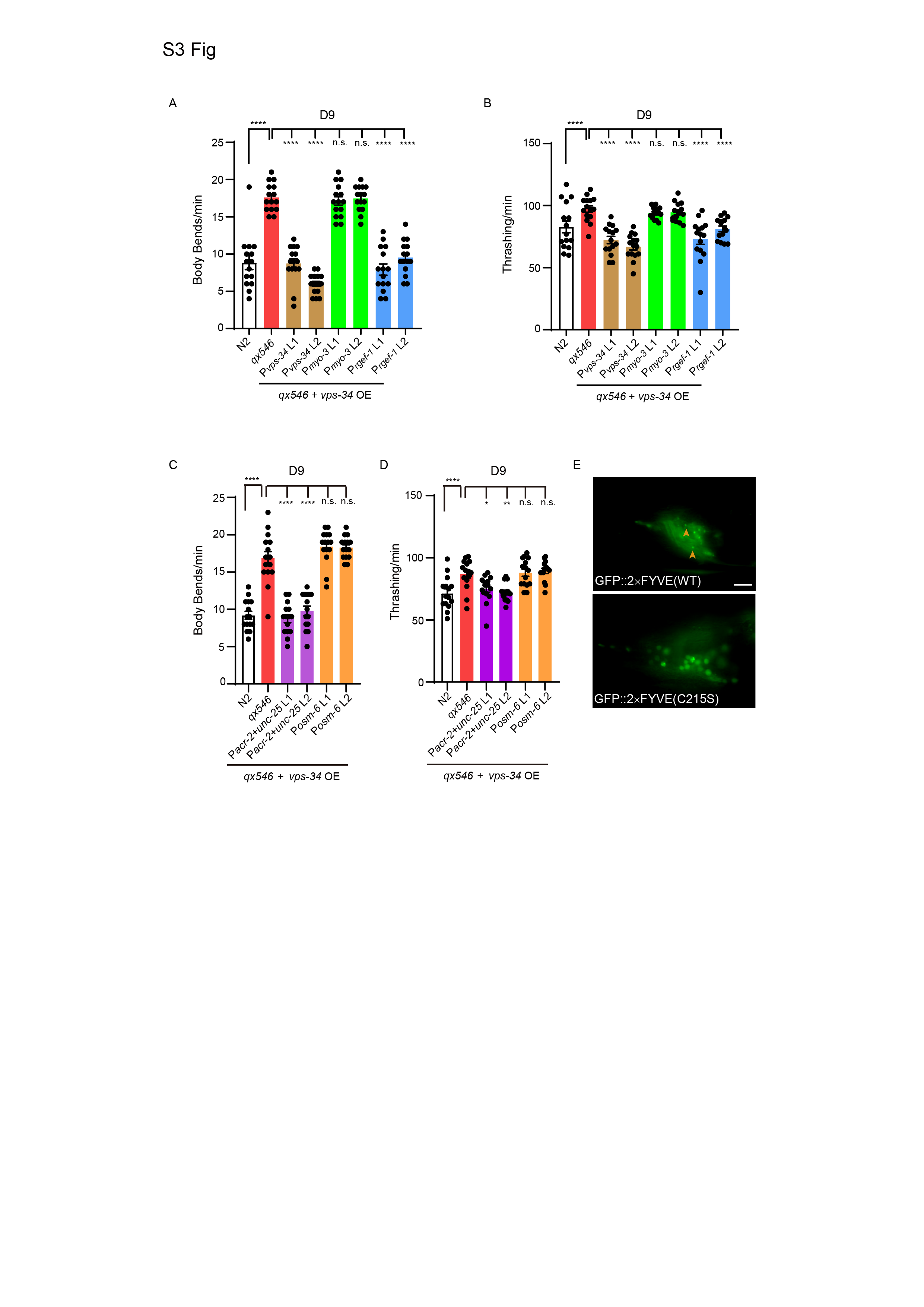

Supplement: S3 Fig — (A and B) Motor behavior assays (Body bends, A; Thrashing, B) comparing N2, qx546, and qx546 overexpressing (OE) vps-34 under vps-34 promoter (Pvps-34), pan-neuronal promoter (Prgef-1), body wall muscle specific promoter (Pmyo-3) at D9. Two independent transgenic lines (L1 and L2) for each genotype were tested. Fifteen worms per group. n = 2 independent experiments. (C and D) Motor behavior assays (Body bends, C; Thrashing, D) comparing N2, qx546, and qx546 overexpressing (OE) vps-34 under motor neuron promoters (Pacr-2+unc-25) or sensory neuron promoter (Posm-6) at D9. Two independent transgenic lines (L1 and L2) for each genotype were tested. Fifteen worms per group. n = 2 independent experiments. (E) Representative images of GFP::2xFYVE and GFP::2xFYVE (C215S) are shown in the nerve ring. Orange arrowheads indicate GFP+ PI(3)P puncta. Scale bar, 10 μm. Error bars, SEM. **P < 0.01, ***P < 0.001, ****P < 0.0001. n.s., not significant. A–D, One-way ANOVA with Dunnett’s multiple comparisons test. Raw data can be found in the Supporting information (S1 Data). (TIF) [file pbio.3002165.s003.tif]

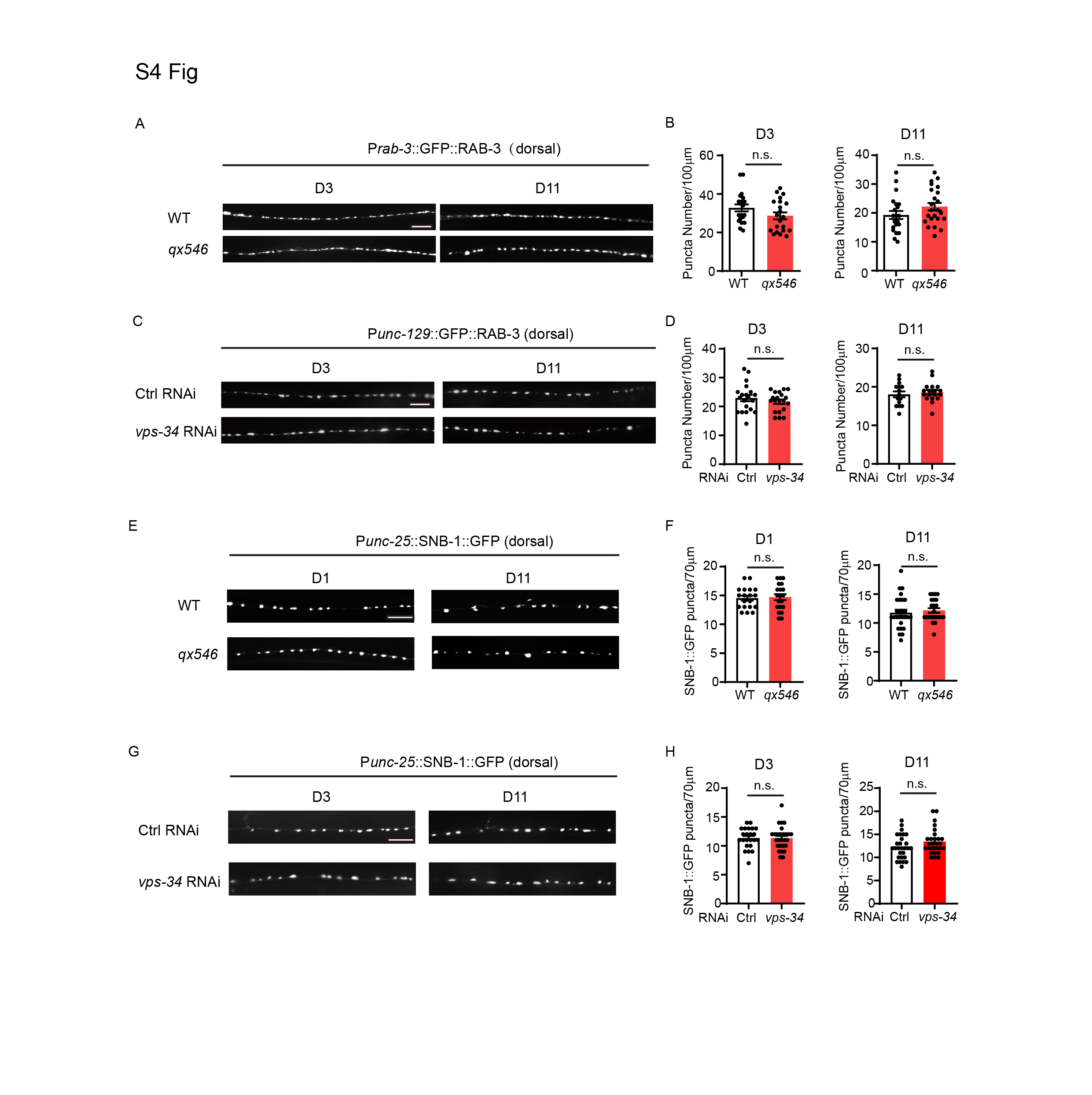

Supplement: S4 Fig — (A) Representative fluorescent images of GFP::RAB-3 in the dorsal nerve cord axons from WT or qx546 worms at D3 and D11. (B) Quantification of GFP::RAB-3 labeled synaptic puncta in (A). (C) Representative fluorescent images of GFP::RAB-3 under unc-129 promoter in the dorsal nerve cord axons of excitatory neurons from ctrl or vps-34 RNAi worms at D3 and D11. (D) Quantification of GFP::RAB-3 labeled synaptic puncta in (C). (E) Representative fluorescent images of SNB-1::GFP under unc-25 promoter in the dorsal nerve cord axons of inhibitory neurons from WT or qx546 worms at D1 and D11. (F) Quantification of SNB-1::GFP labeled puncta in (E). (G) Representative fluorescent images of SNB-1::GFP under unc-25 promoter in the dorsal nerve cord axons from ctrl or vps-34 RNAi worms at D3 and D11. (H) Quantification of SNB-1::GFP labeled puncta in (G). A–H, Over 15 worms per group. n = 2 independent experiments. Scale bar, 10 μm. Error bars, SEM. **P < 0.01, ***P < 0.001, ****P < 0.0001. n.s., not significant. All other points as P > 0.05. B, D, F, H, Unpaired two-tailed Student’s t test. Raw data can be found in the Supporting information (S1 Data). (TIF) [file pbio.3002165.s004.tif]

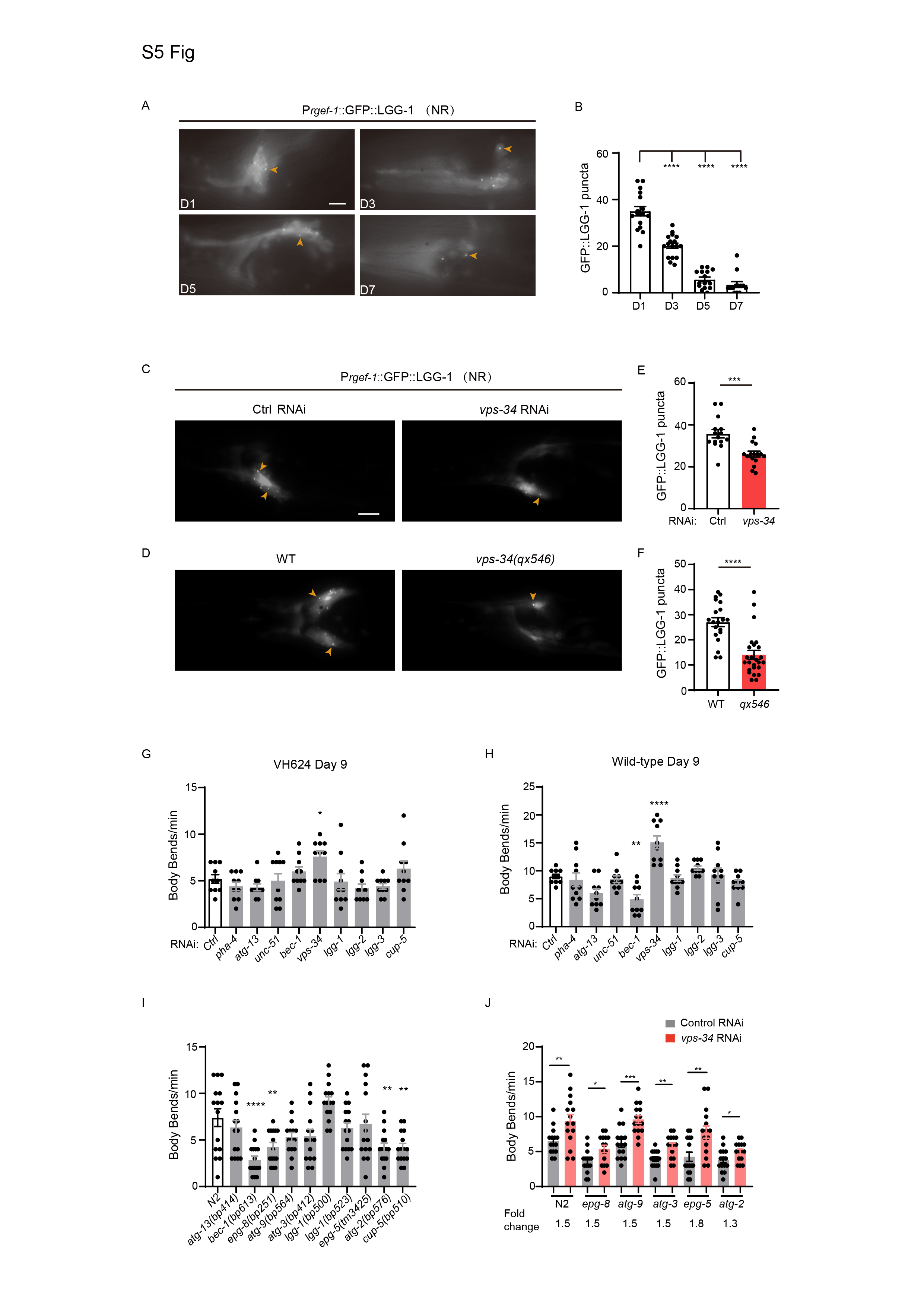

Supplement: S5 Fig — (A) Representative fluorescent images showing the GFP::LGG-1 in the NR during aging. Orange arrowheads indicate GFP::LGG-1 labeled puncta. (B) Quantification of GFP::LGG-1 labeled puncta numbers in the NR. Fifteen worms per group. n = 3 independent experiments. (C and D) Representative fluorescent images of GFP::LGG-1 in the NR from Ctrl RNAi and vps-34 RNAi (C) or WT and vps-34(qx546) (D). (E and F) Quantification of GFP::LGG-1 puncta numbers in Ctrl RNAi, vps-34 RNAi, WT and vps-34(qx546) nerve ring. Fifteen worms per group. n = 2 independent experiments. (G and H) Motor behavior assays (Body bends) comparing ctrl and autophagy-related gene RNAi groups. (G) VH624 strain was used. (H) wild-type strain was used. (I) Frequency of body bends was quantified in autophagy mutants compared to N2. (J) Frequency of body bends was quantified in N2 or autophagy mutants with vps-34 KD. The fold changes of vps-34 RNAi vs. control RNAi in each group were calculated: wild-type (1.5-fold) and other autophagy mutant such as epg-8 (1.5-fold), atg-9 (1.5-fold), atg-3 (1.5-fold), epg-5 (1.8-fold), atg-2 (1.3-fold). G and H, 10 worms per group. I and J, 15 worms per group. n = 2 independent experiments. Error bars, SEM. **P < 0.01, ***P < 0.001, ****P < 0.0001. n.s., not significant. All other points as P > 0.05. B, E, F, J, Unpaired two-tailed Student’s t test. G–I, One-way ANOVA with Dunnett’s multiple comparisons test. Scale bars, 10 μm. Raw data can be found in the Supporting information (S1 Data). (TIF) [file pbio.3002165.s005.tif]

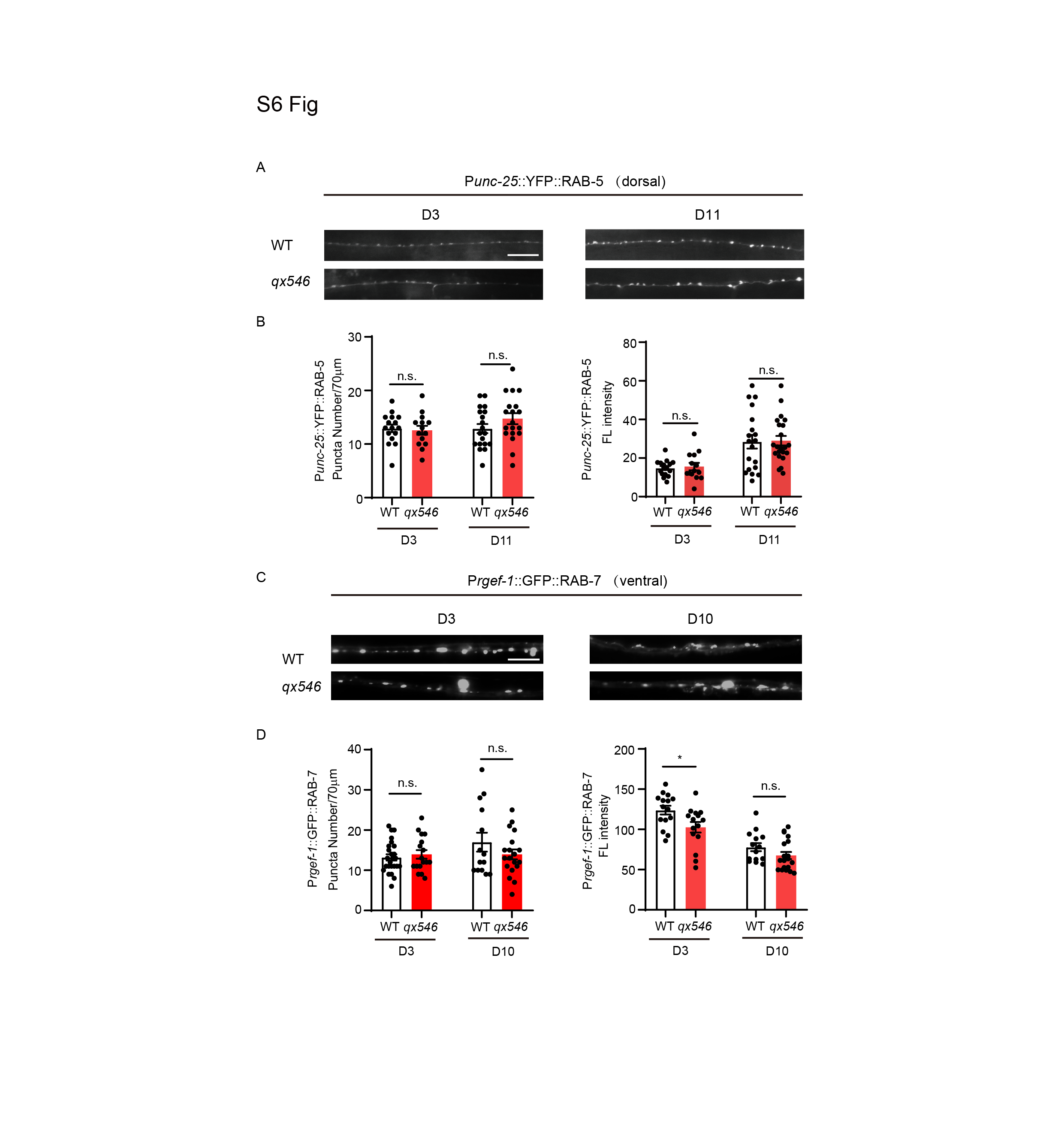

Supplement: S6 Fig — (A) Representative fluorescent images of YFP::RAB-5 under unc-25 promoter in the dorsal nerve cord inhibitory neurons in young (D3) and aged (D11) WT or qx546 worms. (B) Quantification of YFP::RAB-5 puncta number and intensity in D3 and D11 WT or qx546 worms. (C) Representative fluorescent images of GFP::RAB-7 in the ventral nerve cord in young (D3) and aged (D10) WT or qx546 worms. (D) Quantification of GFP::RAB-7 puncta number and intensity in D3 and D10 WT or qx546 worms. A–D, Over 15 worms per group. n = 2 independent experiments. Error bars, SEM. *P < 0.05, **P < 0.01, ***P < 0.001, ****P < 0.0001. n.s., not significant. B, D, Unpaired two-tailed Student’s t test. Raw data can be found in the Supporting information (S1 Data). (TIF) [file pbio.3002165.s006.tif]

Raw immunoblots

Related to S2A Fig

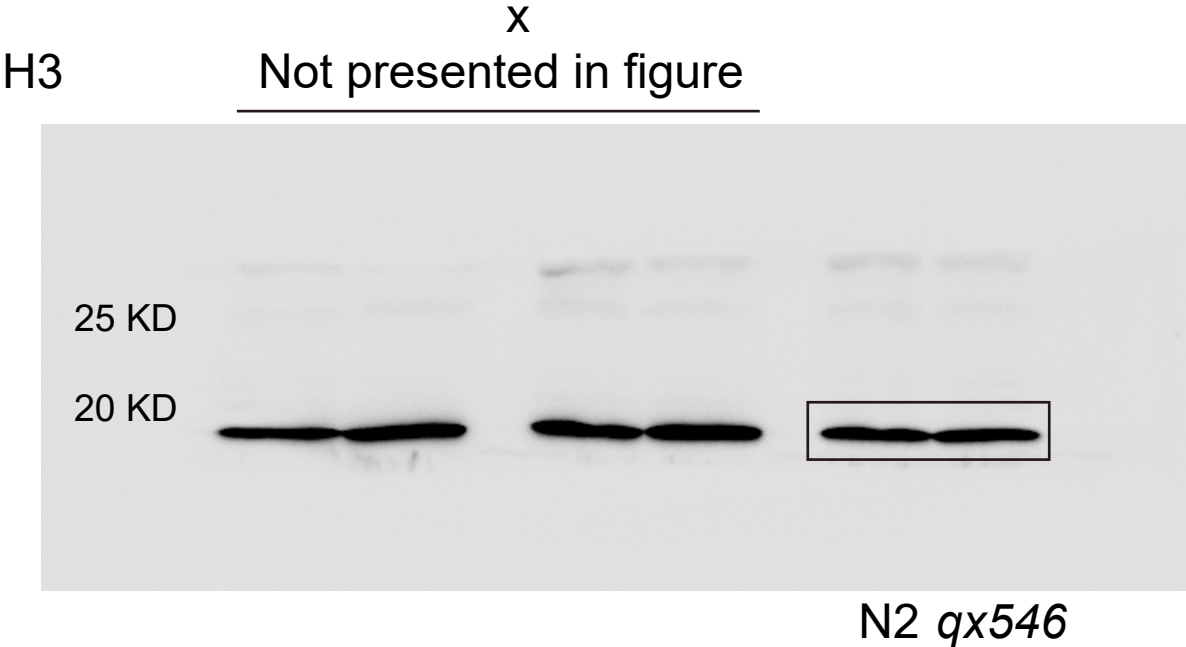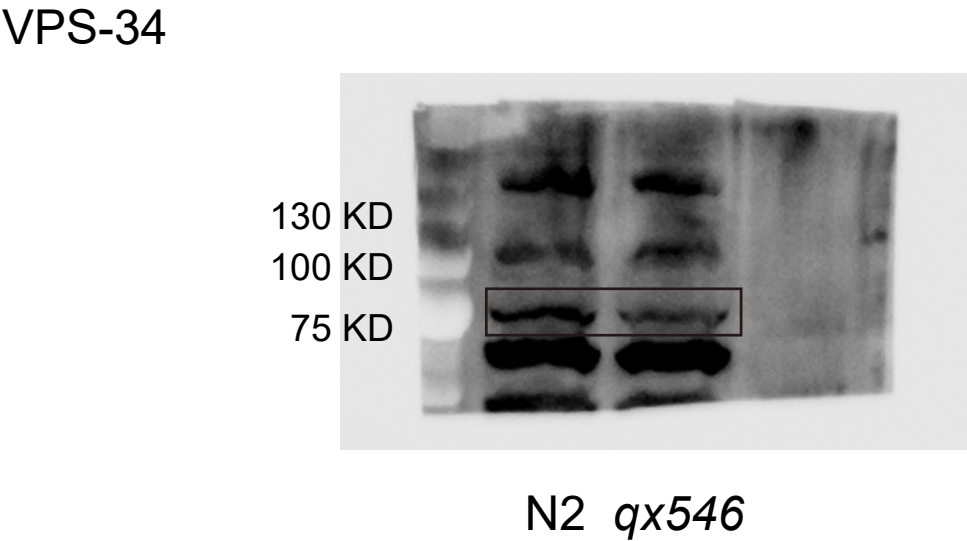

Supplement: S1 Raw Images — (PDF) [file pbio.3002165.s014.pdf]
