# Supplementary material for: Partial inhibition of class III PI3K VPS-34 ameliorates motor aging and prolongs health span
Source: PLoS Biol. 2023 Jul 11;21(7):e3002165. doi: 10.1371/journal.pbio.3002165 (PMC10335676; doi:10.1371/journal.pbio.3002165)
Supplement: Attachment — Submitted filename: Response to reviewers.docx [file pbio.3002165.s015.docx]

Response to Reviewers' Comments (PBIOLOGY-D-22-02899R1)

We would like to thank both reviewers for their high-quality reviews. Their critiques have greatly helped to improve the manuscript. Following their constructive suggestions, we have substantially revised our manuscript and figures.

Below, we provide a point-by-point rebuttal to the issues they raised.

**Reviewer #1** writes: *“In this work, Hu et al. performed a novel RNAi screen to identify genes whose inactivation allows worms to maintain mobility in old age. They identified and further characterized the role of one of the candidate genes (out of a total of 34) that encodes the class III Phosphatidylinositol 3-kinase, VPS-34. Genetic and electrophysiological approaches led them to conclude that VPS-34 reduces neurotransmission in aged neurons via inhibition of the conversion of PI3P to PI4P. The use of a drug targeting this kinase in mice leads to an increase in the size of muscle fibres and a change in their metabolism at an advanced age.*

*These results are interesting because the molecular actors responsible for motorneurons aging in C.elegans are largely unknown. The authors used multiple approaches relevant to the characterization of their phenotype, but some results are still preliminary and need further substantiation.”*

Response: We thank this reviewer for his/her summary and positive comments.

Major concerns:

1. *“the number of worms counted for the motility tests is insufficient (double counting and blind-scoring are required)”*

Response: We agree with the reviewer’s assessment, but we’d like to point out we did perform blind-scoring for the motility tests in our previous submission (detailed in the Methods section). While we did not perform double counting, we performed independent experiments to repeat our scoring (each repeat detailed in Source Data), and two independent behavioral tests (body bends on plates and thrashing in medium) to confirm our results. To further strengthen our point as the reviewer suggested, we re-performed some of the key behavioral experiments on a larger cohort of worms (30-50 worms per group) using blind-scoring, including *vps-34* RNAi versus control, *qx546* versus N2, and SAR405 versus vehicle treatment during aging (**revised Fig. 1D and E**, **Fig. 1H and I, Fig. 4C and D**). The results are consistent with those in our previous submission.

2. *“in the current manuscript, PI3P or PI4P scoring is based on poor quality and unconvincing images of the nerve ring (which vesicle size was considered? in which ROI (region of interest); why not always make the measurements in the nerve cord as in Figure 3 L/M (in which the number of dots should be given relative to the length of the cord)”*

Response: We thank the reviewer for pointing out the problems with our PI3P or PI4P scoring. To address the image quality issues, we reperformed the experiments and captured the image using confocal microscope. As the reviewer suggested, we respectively performed PI3P or PI4P scoring within the same length of the ventral nerve cord, from the vulva to the tail part of the cord (ROI) (**revised Fig. 3J-L, Fig. 4A and B**). To exclude false positives, individual vesicles greater than 0.2 μm in size were counted (**Revised methods, Line 533**). The results are consistent with those in our previous submission. Since the PI3P puncta is very prominent in the nerve ring, we kept one independent quantification of PI3P in the nerve ring comparing WT and *qx546* worms using recaptured images **(revised Fig. 2H and I)**, with a ROI between the first pharyngeal bulb and terminal pharyngeal bulb.

3. *“In dual RNAi experiments (Figure 3D) the control should be vps-34 RNAi mixed 1:1 with HT115 control bacteria rather than concentrated vps-34 RNAi”*

Response: We apologize for this confusion. We did use vps-34 RNAi mixed 1:1 with HT115 control bacteria but did not mention it in the previous submission. We revised the figure legends to emphasize on this point (**Fig. 3D-I**).

4. *“Normalisation of RT-QPCR data should use 3 control genes instead of one.”*

Response: Following the reviewer’s suggestion, we reperformed the RT-PCR experiment using 4 control genes *act-1, tba-2, ama-1,* and *cdc-42* (**Methods, Line 455**). We found *vps-34* mRNA level is consistently reduced by about 65% in *vps-34* RNAi group compared to the control RNAi group (**Line 124**, **revised Fig. S1D**).

5. *“What is the impact of the vps-34 mutation on longevity?: no values and statistics are reported for figure S2G”*

Response: We have added the p-value and statistics on **Fig. S2G**. *vps-34(qx546)* mutation moderately increases the median lifespan by about 17%, which is statistically significant. The survival curve is representative of three independent repeats, listed in **Table S3**.

6. “*Figure S5: the presentation of the results should compare the effect of vps-34 RNAi in a wild-type or mutant background (rather than control RNAi or vps-34 in a mutant background)”*

Response: Following the reviewer’s suggestion, we compared the effects of vps-34 RNAi in a wild-type or mutant background. Since some of the autophagy mutants exhibited apparently reduced basal level motility, we calculated the fold changes of *vps-34* RNAi vs control RNAi in each group, and compared the rescue effect of *vps-34* RNAi in a wild-type or autophagy mutant. We observe similar levels of rescue effects across different autophagy mutant background: wild-type (1.5-fold) and other autophagy mutant such as *epg-8* (1.5-fold), *atg-9* (1.5-fold), *atg-3* (1.5-fold), *epg-5* (1.8-fold), *atg-2* (1.3-fold) (**Line 327**, **revised Fig. S5J**), consistent with our previous conclusion that VPS-34-regulated autophagy does not play a major role in motor aging.

7. *“the legend in figure 3H is incomplete (strain? age?)”*

Response: We have added the strain and age information in the revised figure legend.

8. *“a thrashing test for all candidates to confirm their phenotype would be welcome”*

Response: We did perform thrashing tests for all candidates. RNAi of 30/34 candidate genes exhibited varying degrees of improvement of thrashing frequency in aged worms (7 increased the motility by >100%, 20 by 20%-100%, and 3 by 5-20%) (**Line 114**), supporting the robustness of our screening method. Since our studies on some of these genes are either under review elsewhere or currently ongoing, we regret we can’t provide a detailed list of these results due to conflict of interest concerns.

**Reviewer #2** writes: *“In this manuscript, the authors established a simple and handy paradigm to assess motor ability in aging C. elegans. By performing genome-wide RNAi screening, the authors identified 34 genes that contribute to motor aging. One of the top hits, Class III PI3K VPS-34, modulates motor aging by regulating neurotransmission in motor neurons and the integrity of muscles. Genetic and* *pharmacological inhibition of VPS-34 with SAR405 ameliorate age-related motor decline in both worms and mice. In this study, Zhang and her colleges provide a unique platform to explore motor aging, and find a novel mechanism underlying motor aging. The conclusions in this study are compelling, and the findings are of high significance in field of motor aging. There are some minor issues.”*

Response: We thank this reviewer for these positive comments.

1. *“It's interesting that partial inhibition rather than complete blockade of VPS34 could improve motor ability. Are there any deleterious effects with VPS-34 inhibition?”*

Response: We carefully examined the worms from *vps-34* RNAi, *qx546*, and SAR405 partial inhibition groups, and did not observe deleterious effects in terms of brood size, overall development, and lifespan (**Line 401**).

2. *“VPS34 is the only class III PI3K regulating the PI(3)P level and it may be required in multiple organs or cell types, so whether the inhibition also has protective effect on other organs?”*

Response: We fully agree. Indeed, we also examined the effects of VPS-34 partial inhibition on multiple other organs, including the epidermis, intestine, and pharynx. In the epidermis of aged worms, the number of vesicular lysosomes is reduced but the volume of each lysosome was increased compared to young worms, which is characteristic of age-related lysosome dysfunction (**Methods, Line 507**). These phenotypes are consistently rescued by *vps-34(qx546)* mutation and SAR405 inhibition (**Line 176**, **revised Fig. S2J-O**). The intestinal atrophy is also a prominent aging feature that is closely related to the lifespan, characterized by reduced relative intestinal width (**Methods, Line 516**). *vps-34(qx546)* mutation consistently increased the relative intestinal width on D9 (**Line 181**, **revised Fig. S2P**). Finally, we examined the pharyngeal pumping rate (**Methods, Line 511**), which also declines with aging, but did not observe significant rescue effect of *qx546* mutation in aged worms (**Line 184, revised Fig. S2Q**). Thus, partial inhibition of VPS-34 appears to show protective effects on the intestine and epidermis, but not pharynx in aged *C. elegans*. Given the importance of VPS-34 function, it is possible that it may have protective effects on additional organs, which could be examined in future analysis.

3. *“What's the physiological function of VPS-34, and whether it's function is age-dependent?”*

Response: Our working model is that VPS-34-regulated PI(4)P-PI-PI(3)P conversion, but not autophagy and endocytosis, underlies motor aging. To test whether this process is age dependent, we calculated the ratio of PI4P versus PI3P using reporter strains, and found that it does decline with aging in control worms, but could be rescued by *vps-34* RNAi (**Line 302**, **revised Fig. 3M**).

4. *“The data showed no significant difference in D5 and D7 RNAi on delaying motor decline, is there a key time point to inhibit the VPS-34?”*

Response: To test whether there is a key time point to inhibit VPS-34, we performed additional *vps-34* RNAi from D9 (old-age). While we did not observe a significant improvement on D11 and D13, at later time points (D15 and D17), *vps-34* RNAi improved the thrashing and/or body bend (**Line 136**, **revised Fig. S1I and J**). In contrast, D5 or D7 (mid-age) RNAi has a more immediate and long-lasting rescue effect. Thus, while VPS-34 inhibition from both mid-age and old-age are beneficial to delay motor aging, it appears that inhibition no later than mid-age has more dramatic effect.
